# Effects of long-term nitrogen & phosphorus fertilization on soil microbial, bacterial and fungi respiration and their temperature sensitivity on the Qinghai-Tibet Plateau

**DOI:** 10.7717/peerj.12851

**Published:** 2022-02-24

**Authors:** Kelu Chen, Huakun Zhou, Yang Wu, Ziwen Zhao, Yuanze Li, Leilei Qiao, Guobin Liu, Sha Xue

**Affiliations:** 1 State Key Laboratory of Soil Erosion and Dryland Farming on the Loess Plateau,Institute of Soil and Water Conservation, Northwest A&F University, Yangling, China; 2 Qinghai Provincial Key Laboratory of Restoration Ecology in Cold Regions, Northwest Institute of Plateau Biology, Chinese Academy of Sciences, Xining, China; 3 Qinghai University, Xining, China; 4 Institute of Soil and Water Conservation, Chinese Academy of Sciences and Ministry of Water Resources, Yangling, China; 5 University of Chinese Academy of Sciences, Beijing, China

**Keywords:** SOC decomposition, Fertilization, SOC quality, Tibetan Plateau

## Abstract

**Background:**

The microbial decomposition of soil organic carbon (SOC) is a major source of carbon loss, especially in ecologically fragile regions (*e.g.,* the Tibetan Plateau), which are also affected by global warming and anthropogenic activities (*e.g.,* fertilization). The inherent differences between bacteria and fungi indicate that they are likely to play distinct roles in the above processes. However, there still have been no reports on that, which is restricting our knowledge about the mechanisms underlying SOC decomposition.

**Methods:**

A long-term nitrogen (N) and phosphorus (P) addition field experiment was conducted to assess their effects on soil microbial, fungal, and bacterial respiration (RM, RF, and RB, respectively) and temperature sensitivity (Q10; at 15 °C, 25 °C, and 35 °C) using cycloheximide and streptomycin to inhibit the growth of fungi and bacteria.

**Results:**

We found that N suppressed RM and RF at all temperatures, but RB was only suppressed at 15 °C, regardless of the addition of P. The addition of N significantly decreased the ratio of RF/RM at 35 °C, and the combined NP treatment increased the Q10 of RB but not that of RF. Results of the redundancy analysis showed that variations in soil respiration were linked with NO_3_^−^-N formation, while the variations in Q10 were linked with SOC complexity. Long-term N addition suppressed RM by the formation of NO_3_^−^-N, and this was mediated by fungi rather than bacteria. The contribution of fungi toward SOC decomposition was weakened by N addition and increasing temperatures. Combined NP addition increased the Q10 of RB due to increased SOC complexity. The present study emphasizes the importance of fungi and the soil environment in SOC decomposition. It also highlights that the role of bacteria and SOC quality will be important in the future due to global warming and increasing N deposition.

## Introduction

Soil microbial respiration (RM) is a key process that drives carbon (C) emissions from underground ecosystems into the atmosphere (Bond-Lamberty & Thomson, 2010). A small change in RM could consequently have a notable impact on the global C cycle. The temperature sensitivity (Q10) of RM is an important parameter in the models that are used to extrapolate the potential effects of climate change. The enzyme kinetics hypothesis proposes that Q10 is negatively related to substrate quality (*i.e.,* the C quality-temperature hypothesis), and this has been supported by both experimental and modeling studies (Fierer et al., 2005; Davidson & Janssens, 2006; Hartley & Ineson, 2008; Craine et al., 2013; Wagai et al., 2013); however, contradictions to the hypothesis have also been reported (Fang, Smith & Smith, 2005; Rey & Jarvis, 2006). Accumulating evidence has also shown that RM and Q10 are not constant but are affected by multiple factors, such as the soil temperature, moisture, and nutrition level as well as its microorganisms (Von Lützow & Kögel-Knabner, 2009; Davidson & Janssens, 2006; Wang et al., 2017).

Nitrogen (N) inputs caused by N deposition and fertilization can alter the quantity and quality of soil organic matter (SOM) (Shahbaz et al., 2017; Zang et al., 2017; Wen et al., 2019a; Wen et al., 2019b; Zang et al., 2020) as well as the associated microbial communities (Dai et al., 2018), all of which can affect RM and its Q10 (Guo et al., 2017). Alterations in the levels of available nutrients due to fertilization can also affect microbial C use efficiency (Zang et al., 2017). The Q10 of the labile C pools is usually lesser than (Xu et al., 2010; Lefevre et al., 2014) that of the recalcitrant pools, although opposite (Fierer et al., 2005) and neutral effects (Fang, Smith & Smith, 2005) have also been detected. Thus, the absolute levels of labile and recalcitrant pools or their ratios are changed due to fertilization and can drive the Q10 of SOM. However, the effects of N addition on Q10 remain controversial (Li et al., 2019; Xiao et al., 2020). In addition, phosphorus (P) deposition have become an important source of P globally (Ahn & James, 2001). A previous study showed that there was no significant influence of P addition on the Q10 of soil microbial respiraiton, but the addition of excess P in the N + P treatment increased Q10 (Wang et al., 2017). These results suggest that the effect of P on Q10 may be dependent on N availability. Furthermore, a long-term experiment conducted in a Tibetan alpine meadow showed that N + P treatment increased Q10 (Guo et al., 2017), which was in contrast to the suppressive effect of short-term N + P treatment on Q10 in the Chinese Loess Plateau (Wang et al., 2017). Therefore, a site-specific experiment is currently required to further investigate the effects of the nutritional inputs on Q10, given its high spatial (Li et al., 2018) and temporal (Yu et al., 2020) heterogeneity.

Both heterotrophic fungi and bacteria are major decomposers of SOM in soil ecosystems, but they have many inherent differences, especially in their resource utilization. Bacteria prefer to consume simple C compounds, while fungi compete for more complex compounds. In addition, they differ in terms of their enzymatic capabilities (Boer et al., 2005), biomass turnover rates (Rousk & Bååth, 2011), and C use efficiency (Six et al., 2006), due to the differences in their morphological and physiological traits (Boer et al., 2005; Strickland & Rousk, 2010). In general, fungi are multicellular organisms and are often characterized by their hyphae, which confer benefits to fungi compared to bacteria, as they have the ability to access and use complex materials (Lynd et al., 2002). Several studies have reported that the C:N ratios of plant materials are negatively correlated with the abundance of bacteria in the soil but positively associated with the abundance of fungi (Henriksen & Breland, 1999; Rousk & Bååth, 2007; Poll et al., 2008). In addition, the responses of fungi and bacteria to climate change are different (Yu, Han & Fu, 2019). For example, fungi are more adapted to low temperatures than bacteria (Pietikäinen, Pettersson & Bååth, 2005; Bárcenas-Moreno et al., 2009; Alster, Weller & Von Fischer, 2018). To the best of our knowledge, no previous studies have distinguish between the role of fungi and bacteria in soil respiration and related Q10 responsed to nutrient addition. Such studies, however, could help us to further understand the mechanisms underlying soil organic C (SOC) decomposition and its responses to disturbances, such as climate change.

The Tibetan Plateau is the highest and largest plateau on earth, and the effects of global warming on this region have been markedly more severe than those on other areas, making this plateau an ideal site to study SOM decomposition rates and Q10 values (Li et al., 2018). Given the relative increase in large molecule fractions with sufficient energy to react, the alpine soils are predicted to have larger Q10 values and be at a higher risk of experiencing C losses in response to global warming (Von Lützow & Kögel-Knabner, 2009; Zheng et al., 2009; Wagai et al., 2013). In addition, the Tibetan Plateau has experienced unprecedented anthropogenic disturbances, such as grazing, N deposition, and fertilization (Harris, 2010; Liu et al., 2015). While some studies have investigated the effects of N and P addition on soil C pools and RM and its Q10 on the Tibetan Plateau (Guo et al., 2017; Luo et al., 2019), they have regarded soil microorganisms as a single community ignoring the different roles of bacteria and fungi, which has restricted our knowledge about the mechanisms underlying SOC decomposition. Thus, in the present study, we conducted a long-term N and P addition field experiment on the Tibetan Plateau, using cycloheximide and streptomycin to inhibit the growth of fungi and bacteria, respectively, to separately analyze their respiration and Q10. Redundancy analysis between them and soil physicochemical properties and soil organic carbon complexity(the ratio of recalcitrant carbon in soil organic carbon) to explore the potentially mechanism. We hypothesized the following: (1) N and P addition would suppress soil RM and increase its Q10. (2) Fungal respiration (RF) and its Q10 would be higher than those of bacteria following nutrient addition.

## Materials and Methods

### Study area

The site used for the experiment was near the Haibei Alpine Grassland Ecosystem Research Station (37°36′N; 101°19′E; elevation 3,215 m) of the Northwest Institute of Plateau Biology, Chinese Academy of Science. The climate at the site is characterized by a short, cool summer and long, severely cold winter. The annual mean temperature is −2 °C. The annual mean precipitation is 500 mm, of which >80% occurs during the summer. The plant community is dominated by species, such as *Kobresia humilis*, *Elymus nutans*, and *Festuca ovina*; the soil is Gelic Cambisol (WRB, 1998). A detailed description of this has previously been reported (Chen et al., 2020).

### Experiment design for N and P addition

Four nutrient application blocks were constructed in May 2009. Each block consisted of four 6 m × 6 m plots . For N treatment, N was supplied in the form of urea at a rate of 10 g N m^−2^ y^−1^. For P treatment, P was added as a triple superphosphate at the rate of 5 g P m^−2^ y^−1^. For NP treatment, N and P were co-administered as a combination of urea and triple superphosphate at rates of 10 g N m^−2^y^−1^ and 5 g P m^−2^ y^−1^, respectively. For the control (CK) treatment, the nutrients were supplied at rates simulating those typically applied to the alpine grasslands of the Tibetan Plateau (Jing et al., 2016). The rate of N addition (10 g N m^−2^ y^−1^) was higher than the rate of natural N deposition (Jia et al., 2014).

### Soil sampling and microcosm experimental setup

After surface litter removal, soil cores were collected randomly from the topsoil (0–10 cm) in 2018. Samples from each plot at the same depth were pooled, packed in zip-lock polyethylene bags, stored on ice, transported to the laboratory, and passed through a 2-mm sieve for analyzing soil properties; the methodology of soil and vegetation properties has been reported in detail in a previous study (Chen et al., 2020). The estimate of soil nitrate-nitrogen (NO_3_^−^ -N) and ammonium (NH_4_^+^-N) in fresh soil by using continuous flow autoanalyzer (AutAnalyel, Bran + Luebbe GmbH, Norderstedt, Germany), which were extracted in 1 M KCl (Wu et al., 2021). A liquid TOC II analyzer (Elementar Analyses System, Hanau, Germany) was used to estimate the dissolved organic carbon (DOC) concentration in fresh soil after extraction with distilled water (Jones & Willett, 2006). Before measuring soil properties, soil samples were air-dried and finely ground. The SOC content assay was performed by using the Walkley-Black method (Nelson & Sommers 1982), and soil total nitrogen content was assayed by the Kjeldahl method (Bremner & Mulvaney, 1983). Total phosphorus (TP) content was assayed colorimetrically after H_2_SO_4_ and HClO_4_ digestion. We measured soil pH was measured with a pH meter (1:5 soil to water ratio) (Metrohm 702, Herisau, Switzerland). The belowground biomass of vegetation was collected from soil cores (0–10 cm) in each plot using the soil auger (five cm diameter). All plant roots samples were transferred to the laboratory and oven dried (48 h at 65 °C) then weighed as belowground biomass.

Fungal and bacterial contributions to CO_2_ production were measured by four antibiotic treatments in the microcosm experiment: (1) antibiotic-free control; (2) soil with added streptomycin (2.0 mg g^−1^ soil); (3) soil with added cycloheximide (8.0 mg g^−1^ soil); and (4) soil with added streptomycin and cycloheximide. Bacterial respiratory potential with the soil CO_2_ fluxes was estimated using the equation: A–B; where A is the soil CO_2_ flux in the antibiotic-free control, and B is the CO_2_ flux in the soil treated with streptomycin. This equation was also used to calculate fungal contribution by replacing B with the CO_2_ flux in the soil treated with cycloheximide.

Soil samples (20 g dry weight) were placed in 250-milliliter glass Mason jars and adjusted to 60% water holding capacity (He et al., 2013) by adding deionized water. They were then kept at 25 °C for 7 days in an incubator with automatic temperature and moisture regulation to activate the microorganisms but minimize any disturbance to the “pulse effect”. The glass Mason jars were sealed with parafilm that had small airways to allow for ventilation while still reducing water loss (Fierer & Schimel, 2002). The soil samples were amended with glucose (2 mg g^−1^ soil) and equably sprayed with antibiotic solutions, followed by pre-incubation overnight at 4 °C to ensure the diffusion of antibiotics and glucose into the soil pores. The samples were then incubated at 15 °C, 25 °C, or 35 °C for 7 days to measure their CO_2_ production. For each temperature, there were four fertilization treatments and four field blocks (replications). Each replication included four antibiotic treatments and each of these consisted of three technical repetitions. The average from the three technical repetitions for each antibiotic treatment was calculated for further analysis.

The inhibitor additivity ratio (IAR) was used to determine if the antibiotics exerted non-target effects (*i.e.,* effects on non-target organisms). This was estimated using the following equation: (A−B) + (A−C)/(A−D); where A, B, C, and D represent the CO_2_ fluxes in the antibiotic-free control, streptomycin-amended, cycloheximide-amended, and combined antibiotic-treated soils, respectively. An IAR of ∼1.0 indicated that the inhibitory effects of streptomycin and cycloheximide were not confounded (Beare et al., 1990; Chen, Mothapo & Shi, 2014). We calculated the Q10 values in accordance with the following example, the Q10 of RM: the respiration rates at three different measurement temperatures were used to fit an exponential function according to R = A * e^bT^. Then, Q_10_ was calculated as e^10b^. Here, R was RM at 15 °C, 25 °C, or 35 °C. The Q10 values of soil bacterial respiration (RB) and RF were similar to those of RM (He et al., 2018).

### Gas sampling and measurement

Changes in the slope of CO_2_ production (ppm) were measured in real time using an automatic system (G2301, Picarro, USA) in the dark. Soil RM potential was calculated following He et al. (2013). A detailed description of this seen in Appendix S2.

### Data analysis

Two-way ANOVA (analysis of variance)with Duncan’s multiple range test was performed to detect the effects of the nutrient treatment and elevated temperature on RM, RB, RF, RF/RM, and RB/RM. One-way ANOVA with Duncan’s multiple range test was performed to detect the effect of nutrient treatment on Q10. An independent *t*-test was performed to detect the differences between RF/RM and RB/RM under the same temperature and treatment. Before performing the above-mentioned analysis, the variables that were not satisfied by the assumption of normality were log-transformed. All analyses were performed with IBM SPSS Statistics 22 (IBM, Armonk, NY, USA), and the redundancy analysis (RDA) was conducted using Canoco 5.0 software (Microcomputer Power, Ithaca, NY, USA).

## Results

### Soil microbial, bacterial and fungi respiration

**Figure 1:**
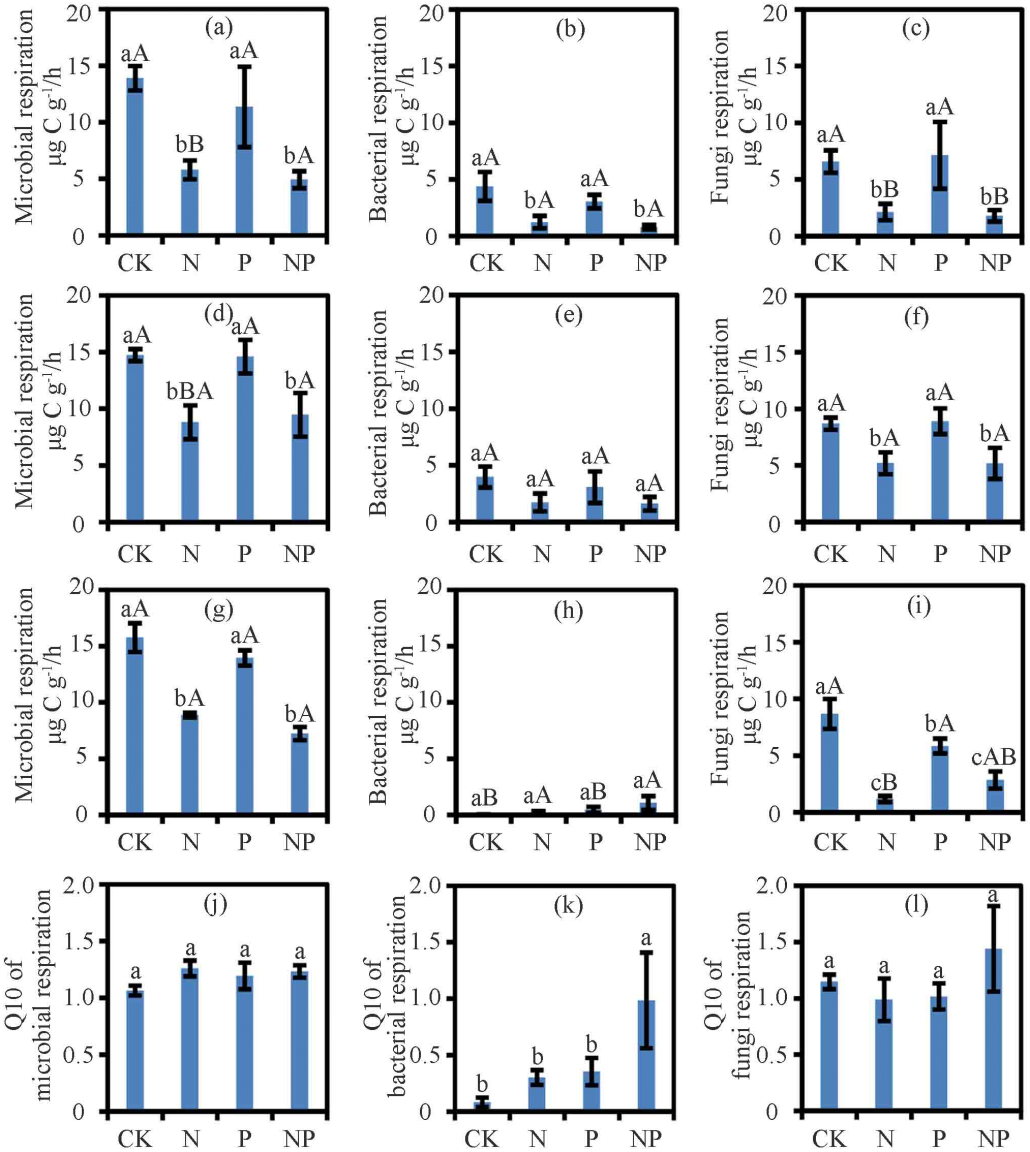
The respiration and Q10 at different treatments and temperatures. Soil microbial respiration rates at 15 °C (A), 25 °C (D) and 35 °C (G), bacterial respiration rates at 15 °C (B), 25 °C (E) and 35 °C (H), fungi respiration rates at 15 °C (C), 25 °C (F) and 35 °C (I) and Q10 of soil microbial respiration (J), soil bacterial respiration (K) and soil fungi respiration (L) are shown. Data are means ± SE. The different lowercase letters above the bars denoted significant differences under control (CK), nitrogen (N) addition, phosphorus (P) addition and nitrogen & phosphorus (NP) addition (*P* < 0.05). The different uppercase letters above the bars denoted significant differences among different temperatures (15 °C, 25 °C and 35 °C) (*P* < 0.05).

Results from the two-way ANOVA showed that nutrient and temperature significantly affected RM, RB, and RF, but the nutrient and temperature interactions only affected RB (Table S1). N and NP treatment suppressed RM and RF at 15 °C, 25 °C, and 35 °C (*P* < 0.05) and RB at 15 °C (*P* < 0.05; Fig. 1). Except for the negative effect on RF at 35 °C (*P* < 0.05; Fig. 1), P treatment had no effect on RF at other temperatures or RM or RB (Fig. 1). With N addition, the RM at 25 °C and 35 °C was higher than that at 15 °C (*P* < 0.05; Fig. 1). With N and P addition, RB and RF at 25 °C were higher than those at 15 °C and 35 °C (*P* < 0.05; Fig. 1).

Nutrient addition, temperature, and their interactions significantly affected the ratio of RF/RM (*P* < 0.05; Table S2). However, in case of ratio of RB/RM was significantly affected by temperature only (*P* < 0.05). At 35 °C, N addition significantly decreased the ratio of RF/RM (*P* < 0.05; Table S3).

### Q10 of soil microbial, bacterial and fungi respiration

The treatments had no effect on the Q10 of RM and RF (Fig. 1). While N and P treatments did not alter the Q10 of RB, their interactions in NP treatment increased the Q10 of RB (*P* < 0.05; Fig. 1).

### Relationship of soil respiration and its Q10 with soil chemical properties and C complexity

**Figure 2:**
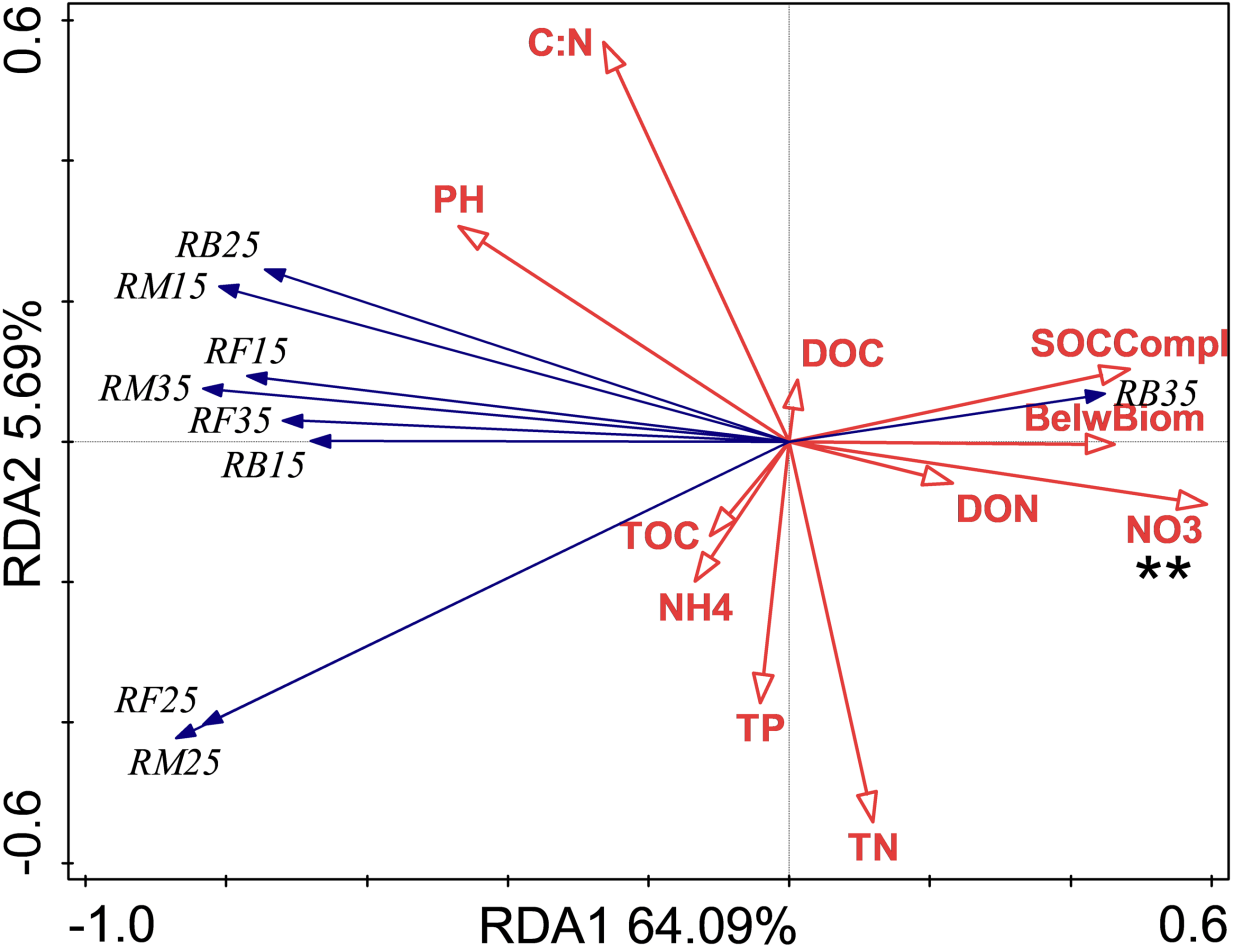
Redundancy analysis (RDA) of respiration at different treatments and temperatures. Soil microbial respiration at 15 °C (RM15), 25 °C (RM25) and 35 °C (RM35), soil bacterial respiration at 15 °C (RB15), 25 °C (RB25) and 35 °C (RB35) and soil fungi respiration at 15 °C (RF15), 25 °C (RF25) and 35 °C (RF35) under control (CK), nitrogen (N) addition, phosphorus (P) addition are shown. BelwBiom, belowground biomass; TO, total organic carbon; TN, total nitrogen; C:N, the ratio of TOC to TON; TP, total phosphorus; PH, pH; DO, dissolved organic carbon; DON, dissolved organic nitrogen; NO_3_, nitrate nitrogen; NH_4_, ammonium nitrogen and SOCCompl, SOC complexity. Two asterisks (**) indicate *P* < 0.05, * indicates *P* < 0.1.

**Figure 3:**
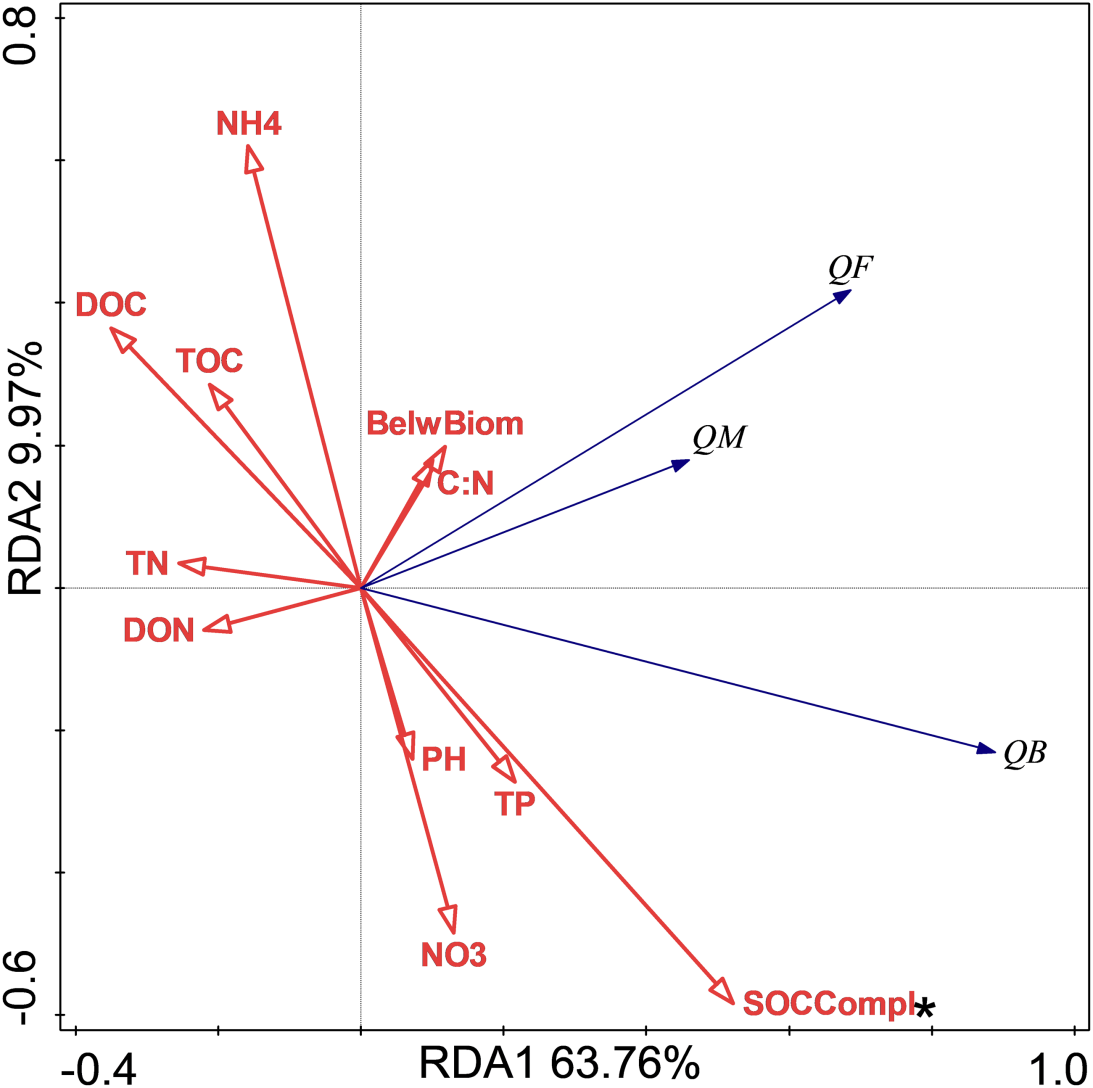
Redundancy analysis (RDA) of Q10 at different treatments and temperatures. The Q10 of soil microbial respiration(QM), Q10 of soil bacterial respiration(QB) and Q10 of soil fungi respiration(QF) are shown. BelwBiom, belowground biomass; TO, total organic carbon; TN, total nitrogen; C:N, the ratio of TOC to TON; TP, total phosphorus; PH, pH; DOC, dissolved organic carbon; DON, dissolved organic nitrogen; NO_3_, nitrate nitrogen; NH_4_, ammonium nitrogen and SOCCompl, SOC complexity. Two asterisks (**) indicate *P* < 0.05, An asterisk (*) indicates *P* < 0.1.

The relationship between soil respiration and its Q10 and soil chemical properties and C complexity are shown in the redundancy analysis results (Figs. 2 & 3). The presented values explained 77.2% of the soil respiration variance (Fig. 2) and 76.1% of the soil respiration Q10 (Fig. 3). NO_3_^−^-N formation could explain 22.9% (*P* < 0.05) of the soil respiration variation (Fig. 2), and SOC complexity could explain 20.8% of the Q10 variation (Fig. 3).

## Discussion

### Response of soil microbial, bacterial and fungi respiration to nutrient addition and temperature

At the adjacent site, nutrient addition for 4 years was found to increase RM, and this was ascribed to increased extracellular enzyme activities and labile SOC levels (Luo et al., 2019). However, after more than 9 years of treatment, N addition was found to suppress RM at all temperatures regardless of P addition. These results support the prediction that N addition initially stimulates and then gradually suppresses RM (Zhou et al., 2014). During short-term fertilization, N addition could accelerate soil RM by: (1) promoting plant growth and thus increasing soil C input (Hoegberg et al., 2006; Zhou et al., 2014); (2) alleviating the N limitations of the soil microorganisms to improve the growth of microbes in the soil and their enzymatic metabolism (Luo et al., 2019); and (3) increasing the labile C stock, most likely through the activation of fresh C to the mineralization of ancient C (Fontaine et al., 2007). In the long-term, nutrient enrichment could alter the plant community composition. Previous studies (Yang et al., 2014a; Yang et al., 2014b) including those conducted at the the same site (Luo et al., 2019; Chen et al., 2020) found that nutrient addition mediated the dominance of community members, because of their different efficiency to take up available nutrients. This may increase the ratio of recalcitrant SOC to labile SOC (Janssens et al., 2010; Guo et al., 2017), making SOM decomposition more difficult. Although the increase in SOC complexity with N addition (data not shown) might support this viewpoint, the redundancy analysis did not identify it as a significant effect. Some studies have also found that N addition can suppress soil RM due to abiotic factors (*e.g.*, acidic soil) (Treseder, 2008). However, the RM did not change with PH responsing to nutrient addition, and we did not identify any significant contributions from other soil physicochemical properties in the redundancy analysis results in the current study. N addition could shift the fungal dominance of the soil microbial community to bacterial dominance (Treseder, 2008; Yuan et al., 2020). After labile SOC that is degraded by the bacteria is exhausted, in accordance with the negative effect of N addition on O-alkyl C levels, as reported by Yuan et al. (2020), microbial growth and related enzyme production would be decreased, SOC would be stabilized in heavier, and RM would gradually be weakened (Neff et al., 2002; Guo et al., 2017). We found that single N addition had negative effects on the ratio of RF/RM but not that of RB/RM. Consequently, shifts in the soil microbial community due to nutrient addition might partially alter respiration. Finally, increased available N levels, implied by a higher NO_3_^−^-N level after N addition in our study (Chen et al., 2020), might suppress microbial gene expression or enzymatic processes related to SOC decomposition. For example, the expression of the ligninolytic enzyme-coding gene (Edwards et al., 2011), oxidative enzyme levels (Cusack et al., 2010), and some N-mining processes, which can degrade SOC as a substrate to obtain N in a N-limited environment, were repressed by N fertilization (Craine, Morrow & Fierer, 2007). This pathway was supported by the results of our redundancy analysis and the increased NO_3_^−^-N content (Chen et al., 2020).

The ratio of RF/RM was higher than that of RB/RM for all treatments at all temperatures (Table S2), suggesting that the suppressive effect of N treatment on RM was mainly mediated by soil fungi. The dominant role of fungi in RM and their responses to nutrient addition might be explained by the differences between the physiological and ecological traits of the fungi and bacteria. Due to the slow biomass turnover rates (Rousk & Bååth, 2011), broad enzymatic capabilities (Boer et al., 2005), and high efficiency for C use (especially complex substrates) (Six et al., 2006) compared with those of bacteria (Lynd et al., 2002), fungi could contribute more to SOC decomposition. Furthermore, owing to strong environmental selection, there were only a few dispersal limitations for the fungi and a more highly connected and positively dominated network compared with those for the bacteria, which could also improve fungal dominance (Xiao et al., 2018). However, at 35 °C, the ratio of RF/RM decreased with N addition, and this was likely due to a decrease in the ratio of plant C:N (Henriksen & Breland, 1999; Rousk & Bååth, 2007; Poll et al., 2008) or a decreased dominance of the fungi in the soil microbial community (Treseder, 2008; Yuan et al., 2020). This indicated that soil N levels increased due to N deposition or fertilization, and this would mean that with future climate warming, the contributions of the fungi to SOC depositions would be weakened.

### The responses the Q10 of soil bacterial respiration to N and P addition

The results showed that only NP treatment increased the Q10 of RB. In general, the Q10 of soil RM was mainly determined by substrate availability, substrate quality, and soil microorganisms (Von Lützow & Kögel-Knabner, 2009), and these factors mediated the effects of biotic factors, such as nutrition, water, and vegetation. The redundancy analysis results showed that only SOC complexity determined the Q10 variations, and our unpublished data also showed that N addition increased SOC complexity. The increase in Q10 value following NP treatment could thus be mediated by soil quality according to the enzyme-kinetics hypothesis (Davidson & Janssens, 2006). In contrast to the Q10 of fungi always being higher than that of bacteria (Alster, Weller & Von Fischer, 2018), NP treatment increased the Q10 of RB rather than that of RF. This could have occurred because increased SOC complexity with nutrient addition was more recalcitrant for bacteria than fungi. In addition, the more difficult the substrate is to decompose, the higher is its Q10 value (Davidson & Janssens, 2006; Wagai et al., 2013).

## Conclusion

Long-term N addition suppressed RM by NO_3_^−^-N formation, which was mediated by fungi rather than bacteria. As the temperature increased, the contribution of fungi to SOC decomposition weakened with the addition of N. NP addition increased the Q10 of RB due to increased SOC complexity. This study not only emphasizes the importance of fungi and soil environment in SOC decomposition, but also highlights the role of bacteria and SOC quality in the future, especially when considering global warming and N deposition.

## Supplemental Information

10.7717/peerj.12851/supp-1 Supplemental Information 1 Supplementary tables

10.7717/peerj.12851/supp-2 Supplemental Information 2 Microbial respiration rate method

10.7717/peerj.12851/supp-3 Supplemental Information 3 Raw data

## Additional Information and Declarations

### Data Availability

The following information was supplied regarding data availability: The raw measurements are available in the Supplemental File.

## References

[ref-1] Ahn H, James RT (2001). Variability, uncertainty, and sensitivity of phosphorus deposition load estimates in south Florida. Water, Air, and Soil Pollution.

[ref-2] Alster CJ, Weller ZD, Von Fischer JC (2018). A meta-analysis of temperature sensitivity as a microbial trait. Global Change Biology.

[ref-3] Bárcenas-Moreno G, Gómez-Brandón M, Rousk J, Bååth E (2009). Adaptation of soil microbial communities to temperature: comparison of fungi and bacteria in a laboratory experiment. Global Change Biology.

[ref-4] Beare MH, Neely CL, Coleman DC, Hargrove WL (1990). A substrate-induced respiration (SIR) method for measurement of fungal and bacterial biomass on plant residues. Soil Biology and Biochemistry.

[ref-5] Bremner JM, Mulvaney CS, Page AL (1983). Nitrogen total. Methods and soil analysis: part 2. Chemical and microbiological properties ASA. Monograph, no. 9.

[ref-6] Boer Wd, Folman LB, Summerbell RC, Boddy L (2005). Living in a fungal world: impact of fungi on soil bacterial niche development. FEMS Microbiology Reviews.

[ref-7] Bond-Lamberty B, Thomson A (2010). Temperature-associated increases in the global soil respiration record. Nature.

[ref-8] Chen H, Mothapo NV, Shi W (2014). The significant contribution of fungi to soil N2O production across diverse ecosystems. Applied Soil Ecology.

[ref-9] Chen W, Zhou H, Wu Y, Wang J, Zhao Z, Li Y, Qiao L, Chen K, Liu G, Xue S (2020). Direct and indirect influences of long-term fertilization on microbial carbon and nitrogen cycles in an alpine grassland. Soil Biology and Biochemistry.

[ref-10] Craine JM, Fierer N, McLauchlan KK, Elmore AJ (2013). Reduction of the temperature sensitivity of soil organic matter decomposition with sustained temperature increase. Biogeochemistry.

[ref-11] Craine JM, Morrow C, Fierer N (2007). Microbial nitrogen limitation increases decomposition. Ecology.

[ref-12] Cusack DF, Torn MS, Mc Dowell WH, Silver WL (2010). The response of heterotrophic activity and carbon cycling to nitrogen additions and warming in two tropical soils. Global Change Biology.

[ref-13] Dai Z, Su W, Chen H, Barberán A, Zhao H, Yu M, Yu L, Brookes PC, Schadt CW, Chang SX (2018). Long-term nitrogen fertilization decreases bacterial diversity and favors the growth of Actinobacteria and Proteobacteria in agro-ecosystems across the globe. Global Change Biology.

[ref-14] Davidson EA, Janssens IA (2006). Temperature sensitivity of soil carbon decomposition and feedbacks to climate change. Nature.

[ref-15] Edwards IP, Zak DR, Kellner H, Eisenlord SD, Pregitzer KS (2011). Simulated atmospheric N deposition alters fungal community composition and suppresses ligninolytic gene expression in a northern hardwood forest. PLOS ONE.

[ref-16] Fang C, Smith P, Smith J (2005). Is resistant soil organic matter more sensitive to temperature than the labile organic matter?. Biogeosciences Discussions.

[ref-17] Fierer N, Craine JM, McLauchlan K, Schimel JP (2005). Litter quality and the temperature sensitivity of decomposition. Ecology.

[ref-18] Fierer N, Schimel JP (2002). Effects of drying–rewetting frequency on soil carbon and nitrogen transformations. Soil Biology and Biochemistry.

[ref-19] Fontaine S, Barot S, Barré P, Bdioui N, Mary B, Rumpel C (2007). Stability of organic carbon in deep soil layers controlled by fresh carbon supply. Nature.

[ref-20] Guo H, Ye C, Zhang H, Pan S, Ji Y, Li Z, Liu M, Zhou X, Du G, Hu F (2017). Long-term nitrogen & phosphorus additions reduce soil microbial respiration but increase its temperature sensitivity in a Tibetan alpine meadow. Soil Biology and Biochemistry.

[ref-21] Harris RB (2010). Rangeland degradation on the Qinghai-Tibetan plateau: a review of the evidence of its magnitude and causes. Journal of Arid Environments.

[ref-22] Hartley IP, Ineson P (2008). Substrate quality and the temperature sensitivity of soil organic matter decomposition. Soil Biology and Biochemistry.

[ref-23] He N, Liu Y, Xu L, Wen X, Yu G, Sun X (2018). Temperature sensitivity of soil organic matter decomposition: new insights into models of incubation and measurement. Acta Ecologica Sinica.

[ref-24] He N, Wang R, Gao Y, Dai J, Wen X, Yu G (2013). Changes in the temperature sensitivity of SOM decomposition with grassland succession: implications for soil C sequestration. Ecology and Evolution.

[ref-25] Henriksen T, Breland T (1999). Nitrogen availability effects on carbon mineralization, fungal and bacterial growth, and enzyme activities during decomposition of wheat straw in soil. Soil Biology and Biochemistry.

[ref-26] Hoegberg P, Fan H, Quist M, Binkley D, Tamm CO (2006). Tree growth and soil acidification in response to 30 years of experimental nitrogen loading on boreal forest. Global Change Biology.

[ref-27] Janssens IA, Dieleman W, Luyssaert S, Subke JA, Reichstein M, Ceulemans R, Ciais P, Dolman AJ, Grace J, Matteucci G, Papale D, Piao SL, Schulze ED, Tang J, Law BE (2010). Reduction of forest soil respiration in response to nitrogen deposition. Nature Geoscience.

[ref-28] Jia Y, Yu G, He N, Zhan X, Fang H, Sheng W, Zuo Y, Zhang D, Wang Q (2014). Spatial and decadal variations in inorganic nitrogen wet deposition in China induced by human activity. Scientific Reports.

[ref-29] Jing X, Yang X, Ren F, Zhou H, Zhu B, He J-S (2016). Neutral effect of nitrogen addition and negative effect of phosphorus addition on topsoil extracellular enzymatic activities in an alpine grassland ecosystem. Applied Soil Ecology.

[ref-30] Jones DL, Willett VB (2006). Experimental evaluation of methods to quantify dissolved organic nitrogen (DON) and dissolved organic carbon (DOC) in soil. Soil Biology and Biochemistry.

[ref-31] Lefevre R, Barre P, Moyano FE, Christensen BT, Bardoux G, Eglin T, Girardin C, Houot S, Kaetterer T, Van Oort F (2014). Higher temperature sensitivity for stable than for labile soil organic carbon–evidence from incubations of long-term bare fallow soils. Global Change Biology.

[ref-32] Li Q, Song X, Chang SX, Peng C, Xiao W, Zhang J, Xiang W, Li Y, Wang W (2019). Nitrogen depositions increase soil respiration and decrease temperature sensitivity in a Moso bamboo forest. Agricultural and Forest Meteorology.

[ref-33] Li J, Yan D, Pendall E, Pei J, Noh NJ, He J-S, Li B, Nie M, Fang C (2018). Depth dependence of soil carbon temperature sensitivity across Tibetan permafrost regions. Soil Biology and Biochemistry.

[ref-34] Liu Y, Wang Y, Pan Y, Piao S (2015). Wet deposition of atmospheric inorganic nitrogen at five remote sites in the Tibetan Plateau. Atmospheric Chemistry & Physics.

[ref-35] Luo R, Fan J, Wang W, Luo J, Kuzyakov Y, He J-S, Chu H, Ding W (2019). Nitrogen and phosphorus enrichment accelerates soil organic carbon loss in alpine grassland on the Qinghai-Tibetan Plateau. Science of the Total Environment.

[ref-36] Lynd LR, Weimer PJ, Van Zyl WH, Pretorius IS (2002). Microbial cellulose utilization: fundamentals and biotechnology. Microbiology and Molecular Biology Reviews.

[ref-37] Neff JC, Townsend AR, Gleixner G, Lehman SJ, Turnbull J, Bowman WD (2002). Variable effects of nitrogen additions on the stability and turnover of soil carbon. Nature.

[ref-38] Nelson DW, Sommers LE, Page AL, Miller RH, Keeney DR (1982). Total carbon, organic carbon, and organic matter. Method of soil analysis, part 2.

[ref-39] Pietikäinen J, Pettersson M, Bååth E (2005). Comparison of temperature effects on soil respiration and bacterial and fungal growth rates. FEMS Microbiology Ecology.

[ref-40] Poll C, Marhan S, Ingwersen J, Kandeler E (2008). Dynamics of litter carbon turnover and microbial abundance in a rye detritusphere. Soil Biology and Biochemistry.

[ref-41] Rey A, Jarvis P (2006). Modelling the effect of temperature on carbon mineralization rates across a network of European forest sites (FORCAST). Global Change Biology.

[ref-42] Rousk J, Bååth E (2007). Fungal and bacterial growth in soil with plant materials of different C/N ratios. FEMS Microbiology Ecology.

[ref-43] Rousk J, Bååth E (2011). Growth of saprotrophic fungi and bacteria in soil. Fems Microbiology Ecology.

[ref-44] Shahbaz M, Kuzyakov Y, Maqsood S, Wendland M, Heitkamp F (2017). Decadal nitrogen fertilization decreases mineral-associated and subsoil carbon: a 32-year study. Land Degradation & Development.

[ref-45] Six J, Frey S, Thiet R, Batten K (2006). Bacterial and fungal contributions to carbon sequestration in agroecosystems. Soil Science Society of America Journal.

[ref-46] Strickland MS, Rousk J (2010). Considering fungal: bacterial dominance in soils–methods, controls, and ecosystem implications. Soil Biology and Biochemistry.

[ref-47] Treseder KK (2008). Nitrogen additions and microbial biomass: a meta-analysis of ecosystem studies. Ecology Letters.

[ref-48] Von Lützow M, Kögel-Knabner I (2009). Temperature sensitivity of soil organic matter decomposition—what do we know?. Biology and Fertility of Soils.

[ref-49] Wagai R, Kishimoto-Mo AW, Yonemura S, Shirato Y, Hiradate S, Yagasaki Y (2013). Linking temperature sensitivity of soil organic matter decomposition to its molecular structure, accessibility, and microbial physiology. Global Change Biology.

[ref-50] Wang R, Sun Q, Wang Y, Liu Q, Du L, Zhao M, Gao X, Hu Y, Guo S (2017). Temperature sensitivity of soil respiration: synthetic effects of nitrogen and phosphorus fertilization on Chinese Loess Plateau. Science of the Total Environment.

[ref-51] Wen Y, Zang H, Freeman B, Ma Q, Chadwick DR, Jones DL (2019a). Rye cover crop incorporation and high watertable mitigate greenhouse gas emissions in cultivated peatland. Land Degradation & Development.

[ref-52] Wen Y, Zang H, Freeman B, Musarika S, Evans CD, Chadwick DR, Jones DL (2019b). Microbial utilization of low molecular weight organic carbon substrates in cultivated peats in response to warming and soil degradation. Soil Biology and Biochemistry.

[ref-53] Wu Y, Chen W, Li Q (2021). Ecoenzymatic stoichiometry and nutrient limitation under a natural secondary succession of vegetation on the Loess Plateau, China. Land Degradation & Development.

[ref-54] Xiao X, Liang Y, Zhou S, Zhuang S, Sun B (2018). Fungal community reveals less dispersal limitation and potentially more connected network than that of bacteria in bamboo forest soils. Molecular Ecology.

[ref-55] Xiao H, Shi Z, Li Z, Wang L, Chen J, Wang J (2020). Responses of soil respiration and its temperature sensitivity to nitrogen addition: a meta-analysis in China. Applied Soil Ecology.

[ref-56] Xu X, Zhou Y, Ruan H, Luo Y, Wang J (2010). Temperature sensitivity increases with soil organic carbon recalcitrance along an elevational gradient in the Wuyi Mountains, China. Soil Biology and Biochemistry.

[ref-57] Yang X, Ren F, Zhou H, He J (2014a). Responses of plant community biomass to nitrogen and phosphorus additions in an alpine meadow on the Qinghai-Xizang Plateau. Chinese Journal of Plant Ecology.

[ref-58] Yang Y, Zhou H, Ye X, Yao B, Wang W, Zhao X, Zhang H (2014b). Short-term responses of plant community structure and function to nitrogen, phosphorus and potassium additions in an alpine meadow of Qinghai-Xizang Plateau. Acta Botanica Boreali-Occidentalia Sinica.

[ref-59] Yu C, Han F, Fu G (2019). Effects of 7 years experimental warming on soil bacterial and fungal community structure in the Northern Tibet alpine meadow at three elevations. Science of the Total Environment.

[ref-60] Yu L, Wang H, Wang Y, Zhang Z, Chen L, Liang N, He J-S (2020). Temporal variation in soil respiration and its sensitivity to temperature along a hydrological gradient in an alpine wetland of the Tibetan Plateau. Agricultural and Forest Meteorology.

[ref-61] Yuan X, Qin W, Xu H, Zhang Z, Zhou H, Zhu B (2020). Sensitivity of soil carbon dynamics to nitrogen and phosphorus enrichment in an alpine meadow. Soil Biology and Biochemistry.

[ref-62] Zang H, Blagodatskaya E, Wang J, Xu X, Kuzyakov Y (2017). Nitrogen fertilization increases rhizodeposit incorporation into microbial biomass and reduces soil organic matter losses. Biology and Fertility of Soils.

[ref-63] Zang H, Blagodatskaya E, Wen Y, Shi L, Cheng F, Chen H, Zhao B, Zhang F, Fan M, Kuzyakov Y (2020). Temperature sensitivity of soil organic matter mineralization decreases with long-term N fertilization: evidence from four Q10 estimation approaches. Land Degradation & Development.

[ref-64] Zheng Z-M, Yu G-R, Fu Y-L, Wang Y-S, Sun X-M, Wang Y-H (2009). Temperature sensitivity of soil respiration is affected by prevailing climatic conditions and soil organic carbon content: a trans-China based case study. Soil Biology and Biochemistry.

[ref-65] Zhou L, Zhou X, Zhang B, Lu M, Luo Y, Liu L, Li B (2014). Different responses of soil respiration and its components to nitrogen addition among biomes: a meta-analysis. Global Change Biology.

