# Supplementary material for: Effects of long-term nitrogen & phosphorus fertilization on soil microbial, bacterial and fungi respiration and their temperature sensitivity on the Qinghai-Tibet Plateau
Source: PeerJ. 2022 Feb 24;10:e12851. doi: 10.7717/peerj.12851 (PMC8882332; doi:10.7717/peerj.12851)
Supplement: Supplemental Information 1 [file peerj-10-12851-s001.docx]

Table S1 Two way ANOVA of the effects of nutrient addition and temperature on soil microbial respiration (RM), bacterial respiration (RB) and fungi respiration (RF).

|  | P(RM) | P(RB) | P(RF) |
| --- | --- | --- | --- |
| Nutrient addition | ＜0.001 | 0.045 | ＜0.001 |
| Temperature | 0.002 | ＜0.001 | ＜0.001 |
| Nutrient addition× Temperature | 0.695 | 0.049 | 0.173 |

Table S2 Two way ANOVA of the effects of nutrient addition and temperature on the ratio of soil bacterial respiration (RB) and fungi respiration (RF) to soil microbial respiration (RM)

|  | P(RB/RM(%)) | P(RF/RM(%)) |
| --- | --- | --- |
| Nutrient addition | 0.867 | 0.002 |
| Temperature | ＜0.001 | 0.001 |
| Nutrient addition× Temperature | 0.111 | 0.025 |

Table S3 The ratio of soil bacterial respiration (RB) and fungi respiration (RF) to soil microbial respiration (RM) under control (CK), nitrogen (N) addition, phosphorus (P) addition and nitrogen & phosphorus (NP) addition treatment at 15℃, 25℃ and 35℃. Data are means ± SE.

|  | 15℃ | | 25℃ | | 35℃ | |
| --- | --- | --- | --- | --- | --- | --- |
|  | RB/RM(%) | RF/RM(%) | RB/RM(%) | RF/RM(%) | RB/RM(%) | RF/RM(%) |
| CK | 30.75±7.95aA | 46.81±8.78abA* | 27.50±7.28aA | 58.80±1.82aA* | 0.17±0.10bB | 53.95±4.91aA* |
| N | 19.32±5.59aA | 34.14±7.71bA* | 20.68±9.93aA | 58.39±3.07aA* | 2.18±1.65abB | 13.34±3.19bB* |
| P | 29.48±4.06aA | 58.28±5.26aA* | 19.04±7.61aA | 60.62±2.78aA* | 3.56±2.22abB | 41.54±2.67aB* |
| NP | 17.52±5.46aA | 38.57±9.62abA* | 14.90±5.83aA | 57.33±10.70aA* | 14.34±7.90aA | 41.28±12.88aA* |

The different lowercase letters denoted significant differences under control (CK), nitrogen (N) addition, phosphorus (P) addition and nitrogen & phosphorus (NP) addition (P < 0.05). The different uppercase letters denoted significant differences among different temperatures (15℃, 25℃and 35 ℃) (P < 0.05). * denoted significant differences between RF/RM and RB/RM (P＜0.05).
