# Supplementary material for: Effects of long-term nitrogen & phosphorus fertilization on soil microbial, bacterial and fungi respiration and their temperature sensitivity on the Qinghai-Tibet Plateau
Source: PeerJ. 2022 Feb 24;10:e12851. doi: 10.7717/peerj.12851 (PMC8882332; doi:10.7717/peerj.12851)
Supplement: Supplemental Information 2 [file peerj-10-12851-s002.docx]

***Microbial respiration rate***

Microbial respiration rate was estimated by a 2-day laboratory incubation. One incubation subsample (40 g) from each plot was adjusted to 60% field capacity and then placed at 25 ℃ for 7 days in 500-ml mason jars in the dark for pre-incubation. After the pre-incubation, we measured the changes in the slope of microbial respiration (ppm) (at 08:00, 12:00, and 16:00; three times a day to obtain an accurate calculation of the average daily respiration rate) in real-time using an automatic system (G2301, Picarro, USA) in the dark, which was the real microbial respiration rate of the sample. The soil microbial respiration rate was calculated as follows (He et al., 2013):

R = C × V × α × β/m

where R represents soil microbial respiration rate (µg C g^-1^ h^-1^), C represents the CO_2_ concentration production slope (C = ▲CO_2_/ ▲t (ppm s^-1^), ▲CO_2_ represents the total amount of carbon dioxide concentration produced in ▲t (t_1_-t_0_), V represents the gas tube and incubation jar volume, m represents the weight of soil samples (g), α represents the conversion coefficient of CO_2_ mass (to transform from CO_2_ to C, 44/12), and β represents a time conversion coefficient (to transform from seconds to hours, 1/3600).

*He, N., Wang, R., Gao, Y., Dai, J., Wen, X., Yu, G., 2013. Changes in the temperature sensitivity of SOM decomposition with grassland succession: implications for soil C sequestration. Ecol Evol 3, 5045–5054.*
